# Attenuated Postprandial GLP-1 Response in Parkinson’s Disease

**DOI:** 10.3389/fnins.2021.660942

**Published:** 2021-07-02

**Authors:** Richard A. Manfready, Phillip A. Engen, Leo Verhagen Metman, Gabriella Sanzo, Christopher G. Goetz, Deborah A. Hall, Christopher B. Forsyth, Shohreh Raeisi, Robin M. Voigt, Ali Keshavarzian

**Affiliations:** ^1^Division of Digestive Diseases and Nutrition, Department of Internal Medicine, Rush University Medical Center, Chicago, IL, United States; ^2^Rush Center for Integrated Microbiome and Chronobiology Research, Rush University Medical Center, Chicago, IL, United States; ^3^Department of Neurological Sciences, Rush University Medical Center, Chicago, IL, United States

**Keywords:** Parkinson’s disease, glucagon-like peptide-1, GLP-1, gut-brain axis, enteroendocrine signaling, intestinal microbiota, short chain fatty acids

## Abstract

The incretin hormone glucagon-like peptide 1 (GLP-1) has neuroprotective effects in animal models of Parkinson’s disease (PD), and GLP-1 receptor agonists are associated with clinical improvements in human PD patients. GLP-1 is produced and secreted by intestinal L-cells in response to consumption of a meal. Specifically, intestinal microbiota produce short chain fatty acids (SCFA) which, in turn, promote secretion of GLP-1 into the systemic circulation, from which it can enter the brain. Our group and others have reported that PD patients have an altered intestinal microbial community that produces less SCFA compared to age-matched controls. In this report, we demonstrate that PD patients have diminished GLP-1 secretion in response to a meal compared to their household controls. Peak postprandial GLP-1 levels did not correlate with PD disease severity, motor function, or disease duration. These data provide the scientific rationale for future studies designed to elucidate the role of GLP-1 in the pathogenesis of PD and test the potential utility of GLP-1-directed therapies.

## Introduction

Parkinson’s disease (PD) is an unrelenting, progressive neurodegenerative disease that affects 1–2% of the population over 60 years of age, with incidence rising in recent years (Kalia and Lang, 2015; Marras et al., 2018; Yang et al., 2020). Treatments for PD are primarily focused on managing symptoms by attempting to correct dopamine loss and enhancing dopaminergic signaling, but these treatments do not impact the underlying cellular degeneration (Kalia and Lang, 2015; Armstrong and Okun, 2020; Marras et al., 2020). Therefore, there is a clear unmet need to identify PD-modifying interventions that prevent or delay dopamine loss and halt or slow clinical progression (Obeso et al., 2017; Stoker et al., 2018).

There is interest in the potential neuroprotective role of the incretin hormone glucagon-like peptide 1 (GLP-1) in PD based on compelling evidence *in vitro*, in rodent models, and in humans (Athauda and Foltynie, 2016; Kim et al., 2017; Bayram and Litvan, 2020; Glotfelty et al., 2020; Li et al., 2020; Salles et al., 2020; Zhang et al., 2020; Wang et al., 2021). The GLP-1 receptor agonist Exendin-4 prevents 6-hydroxydopamine (6-OHDA)-induced death of dopaminergic neurons in neuronal culture (Li et al., 2009; Athauda and Foltynie, 2016), and intraventricular administration protects mice from MPTP-induced dopaminergic cell loss and improves motor function in animal models of PD (Li et al., 2009). Follow-up studies demonstrate that systemic administration of Exendin-4 preserves motor function, decreases microglial activation, and inhibits inflammatory cytokine expression induced by MPTP in mice (Kim et al., 2009), rescues nigrostriatal dopaminergic neurons from the effects of 6-OHDA in rats (Bertilsson et al., 2008), and a sustained release formula (PT320) delays progression of PD-like symptoms in a genetic mouse model of PD (Wang et al., 2021). Other GLP-1 receptor-targeting compounds are also effective at modulating PD-like outcomes in rodents. Systemic administration of the GLP-1 receptor agonist NLY01 is neuroprotective in the A53T α-syn transgenic mouse model of α-synucleinopathy-induced neurodegeneration (Yun et al., 2018). Dual agonists of GLP-1 and glucose-dependent insulinotropic polypeptide (GIP) receptors also show promise: DA-JC4 protects against rotenone-induced effects in rats (Li et al., 2020) and DA-CH5 protects against MPTP-induced effects in mice (Zhang et al., 2020). Finally, colonization of mice with bacteria (*Lactococcus lactis*, MG1363) that constitutively produce GLP-1 protects mice from MPTP-induced effects (Fang et al., 2020). These data are corroborated in PD patients, who, when treated with the GLP-1 receptor agonist exenatide, demonstrate improvements in motor function that are sustained beyond the treatment period (Athauda et al., 2017, 2018, 2019). Taken together, these data suggest that GLP-1 may influence PD pathogenesis and progression.

In humans, food consumption induces GLP-1 secretion, a process that is heavily influenced by the intestinal microbiota. Certain groups of bacteria produce short chain fatty acids (SCFA) as metabolic byproducts and these SCFA can stimulate secretion of GLP-1 by binding to transmembrane free fatty acid receptors (FFAR2 and FFAR3) on L-cells (Zhou et al., 2008; Greiner and Backhed, 2016; Gribble and Reimann, 2019). Microbial communities that have a higher abundance of SCFA-producing bacteria will consequently have more capacity for postprandial secretion of GLP-1. Our group and others have demonstrated that the PD microbiome differs from that of age-matched controls by harboring a reduced relative abundance of putative SCFA-producing bacteria and concurrent lower levels of stool SCFA (Keshavarzian et al., 2015; Unger et al., 2016; Sun and Shen, 2018). We hypothesized that reduced SCFA production in PD patients contributes to clinical progression of PD by lowering GLP-1 secretion by L-cells (Balks et al., 1997). Accordingly, in this study we assessed plasma levels of GLP-1 to determine if levels are attenuated in PD subjects compared to age-matched, household controls. Identifying low GLP-1 as a feature of PD opens up new therapeutic opportunities to augment GLP-1 secretion in PD patients via non-pharmacologic microbiota-directed interventions (e.g., diet, prebiotics, and probiotics) to modify disease course and/or improve symptoms.

## Methods

### Subjects

Household control (*n* = 16) and PD (*n* = 19) subjects were recruited from the Rush University Medical Center Movement Disorders Clinic and the Parkinson’s Disease Gastroenterology (PDGI) Clinic which is a special clinic that address the unique gastrointestinal issues that afflict PD patients. Controls were spouses aged 40–80 years who lived in the same household as a PD subject. The study was approved by Rush University Institutional Review Board (IRB) and subjects provided written, informed consent prior to participation.

Inclusion criteria for PD subjects: (1) age between 40 and 80 years, (2) a current diagnosis of PD (UK Brain Bank Criteria (Hughes et al., 1992), Hoehn and Yahr (H&Y) stages 1–4 inclusive. Exclusion criteria for PD subjects: (1) a history of gastrointestinal disease (except for hemorrhoids or mild reflux disease), (2) antibiotic use, probiotic supplement use, or diet change during the previous 12 weeks, (3) NSAID use within the previous 3 weeks, and (4) diabetes. Inclusion criteria for control subjects: (1) age between 40 and 80 years, (2) no history of neurological disorders or neurodegenerative disease, and (3) living in the same household as an enrolled PD subject. Exclusion criteria were the same as those described for PD subjects.

Parkinson’s disease clinical characteristics including severity were assessed via the Unified Parkinson’s Disease Rating Scale (MDS-UPDRS), a well-validated and widely used tool to quantify motor and non-motor complications of PD including multiple domains assessing non-motor experiences of daily living (Part I), motor experiences of daily living (Part II), motor examination (Part III), and motor complications (Part IV) (Goetz et al., 2008). All PD subjects were taking levodopa in addition to other PD medications. Subject characteristics can be found in Table 1.

**TABLE 1:** Subject characteristics.

	**Control**	**PD**	**Test**
**Number**	16	19	n/a
**Sex (*n*, %)**			
Male	4 (25%)	13 (68%)	*p* = 0.03^‡^
Female	11 (69%)	6 (32%)	
Not reported	1 (6%)	0 (0%)	
**Age (years)**			
Average	66.4	66.8	*p* = 0.87^†^
Range	56–80	55–81	
**Race (*n*,%)**			
Caucasian	13 (81%)	18 (95%)	*p* = 0.28^‡^
African-American	1 (6%)	1 (5%)	
Not reported	2 (13%)	0 (0%)	
**BMI**			
Average	26.8	28.0	*p* = 0.68^†^
Range	20–35	20–48	
**Age at PD onset (years)**			
Average	n/a	56.1	n/a
Range	n/a	45–74	
**Disease duration (years)**			
Average	n/a	11.4	n/a
Range	n/a	7–18	
**MDS-UPDRS**			
Average	n/a	17.0	n/a
Range	n/a	0–37	
**H&Y**			
Median	n/a	2	n/a
Range	n/a	2–3	
**Medication (*n*,%)**			
Dopamine precursor	–	19 (100%)	
Dopamine agonists	–	10 (53%)	n/a
Glutamate antagonist	–	8 (42%)	
Anticholinergics	–	2 (11%)	
COMT inhibitors	–	4 (21%)	
MAO-B inhibitors	–	7 (37%)	
Antidepressant	–	1 (5%)	

*^‡^Chi square analysis.*

*^†^Student’s *t*-test.*

### Study Procedures

**FIGURE 1:**
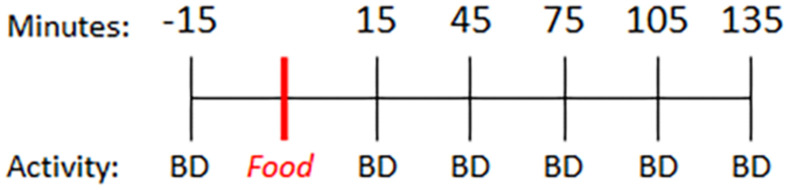
Timeline. Baseline blood sample was collected 15 min prior to consumption of a standard meal (two pieces of toast with butter and coffee) and every 30 min thereafter. BD = blood draw.

Parkinson’s disease subjects were instructed to fast overnight prior to the study visit and take their regular dose of levodopa 3 h prior to initiating this study. On the day of the study visit, PD and control subjects completed questionnaires to assess diet (ASA24^®^) (Subar et al., 2012) and self-reported activity levels. Subjects then underwent a baseline blood collection. Fifteen minutes later, participants consumed a light breakfast consisting of two pieces of toast with butter and coffee (0 min). Blood was collected every 30 min for 4 h (preprandial: −15 min; postprandial: +15, +45, +75, +105, +135 min) (Figure 1). Subjects performed a finger tapping task to quantify motor function at each blood collection time.

Blood was collected via an intravenous line to avoid multiple needle sticks, as previously reported (Swanson et al., 2015). Blood was collected into an EDTA vacutainer tube for plasma separation (#368589; Becton, Dickinson and Company, NJ, United States) and blood was processed within 30 min of each collection. EDTA tubes were centrifuged at 4°C, 3,000 rpm for 15 min. Plasma was aliquoted into cryogenic tubes and stored at −80°C until use. Plasma was used to measure GLP-1 levels using a GLP-1 Total ELISA kit (EZGLP1T-36K; EMD Millipore Corporation) according to the manufacturer’s instructions.

### Data Analysis

Sample size for this study was selected to be powered to detect differences in postprandial GLP-1 levels between control and PD subjects (which at α = 0.5 had a power of >95%). Chi Square Analysis or Student’s *t*-test were used to compare demographic features, diet, and activity level in each group. Student’s *t*-test was used to assess differences between PD and control subjects with respect to preprandial (i.e., fasting) GLP-1 levels. Percent change from baseline to postprandial level was calculated for each subject and percent change in GLP-1 was evaluated using a two-way, repeated measures, mixed effects model ANOVA (factors: time and group). Area under the curve analysis was calculated for each group. A Pearson correlation was used to assess the relationships between GLP-1 and clinical characteristics including motor function (finger tapping), disease severity (MDS-UPDRS), and disease duration. All statistics were performed in GraphPad Prism version 9 (GraphPad Software, LLC).

## Results

### Preprandial GLP-1

**FIGURE 2:**
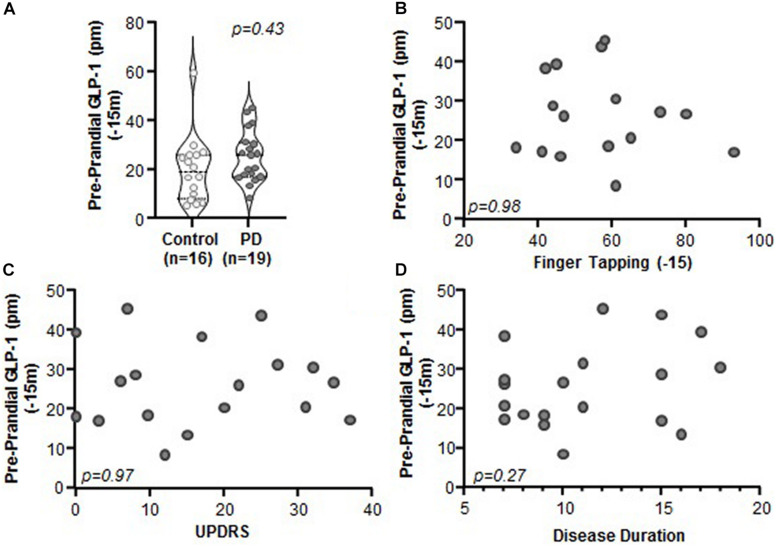
Preprandial GLP-1 levels did not differ between PD and control subjects and did not correlate with PD clinical characteristics. **(A)** Fasting GLP-1 levels were statistically indistinguishable between control and PD subjects (*p* = 0.43). There was no relationship between GLP-1 and clinical characteristics including: **(B)** finger tapping (*p* = 0.98, *r* = 0.005, *r*^2^ < 0.001), **(C)** MDS-UPDRS (*p* = 0.97, *r* = 0.01, *r*^2^ < 0.001), and **(D)** disease duration (*p* = 0.27, *r* = 0.27, *r*^2^ = 0.07).

Comparison of Preprandial (i.e., fasting) levels of GLP-1 between PD and control subjects revealed no significant differences (Figure 2A, *p* = 0.43). Preprandial GLP-1 did not correlate with any of the clinical characteristics that were assessed, including motor function (finger tapping; Figure 2B, *p* = 0.98, *r* = 0.005, *r*^2^ < 0.001), disease severity (MDS-UPDRS: Figure 2C, *p* = 0.97, *r* = −0.01, *r*^2^ < 0.001), or disease duration (Figure 2D; *p* = 0.27, *r* = 0.27, *r*^2^ = 0.07). Lastly, GLP-1 did not correlate with body mass index (BMI) for PD subjects (*n* = 18, *p* = 0.72, *r* = 0.09, *r*^2^ = 0.01) nor for PD and control subjects when combined (*n* = 26, *p* = 0.34, *r* = 0.20, *r*^2^ = 0.04) (data not shown).

### Postprandial GLP-1 Levels

**FIGURE 3:**
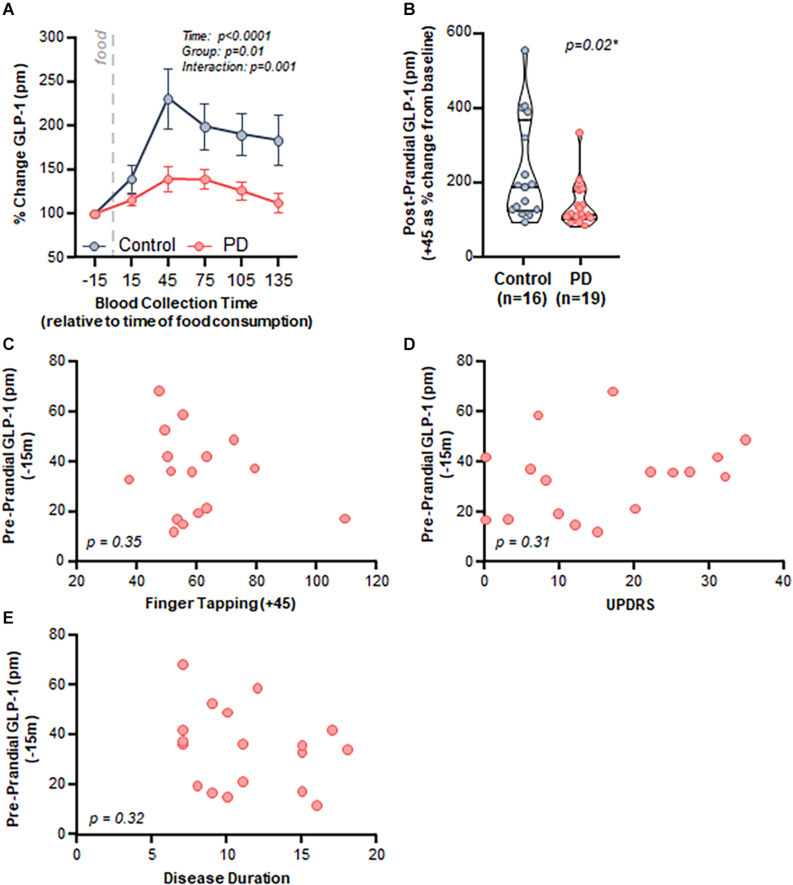
PD patients demonstrated a diminished postprandial GLP-1 response compared to healthy matched control subjects, but there was no relationship with clinical characteristics. **(A)** Time course analysis of GLP-1 revealed a significant effect of time (*p* < 0.0001, *F*_(2__.032__,59__.74__)_ = 15.95), group (Control vs. PD, *p* = 0.01 *F*_(1,33__)_ = 6.90), as well as an interaction (*p* = 0.001 *F*_(5,147__)_ = 4.34). Area under the curve: control = 27,049 ± 4,548, PD = 18,818 ± 2,049, **(B)** Peak postprandial GLP-1 levels were significantly lower in PD subjects compared to controls (*p* = 0.02). There was no relationship between GLP-1 and clinical characteristics including: **(C)** finger tapping (*p* = 0.35, *r* = –0.25, r^2^ = 0.06), **(D)** MDS-UPDRS (*p* = 0.31, *r* = 0.26, *r*^2^ = 0.07), and **(E)** disease duration (*p* = 0.32, *r* = –0.25, *r*^2^ = 0.06).

Both control and PD subjects exhibited an increase in postprandial plasma GLP-1 levels after consumption of a meal; however, the response was attenuated in PD subjects. Specifically, there was a significant effect of time (*p* < 0.001), group (PD vs control, *p* = 0.01), and an interaction (*p* = 0.001) when comparing postprandial GLP-1 response between control and PD subjects (Figure 3A).

Area under the curve analysis revealed that peak GLP-1 levels occurred at + 45 min after consuming a meal (total area ± standard error of the mean: control = 27,049 ± 4,548, PD = 18,818 ± 2,049); therefore, we used the 45-min GLP-1 level for subsequent analyses. GLP-1 percent change from baseline to peak was significantly lower in the PD group compared to the control group (*p* = 0.02, Figure 3B).

Postprandial GLP-1 at 45 min did not correlate with any of the assessed clinical characteristics, including motor function (finger tapping: Figure 3C, *p* = 0.35, *r* = −0.25, *r*^2^ = 0.06), disease severity (MDS-UPDRS: Figure 3D, *p* = 0.31, *r* = 0.26, *r*^2^ = 0.07), and disease duration (Figure 3E, *p* = 0.32, *r* = −0.25, *r*^2^ = 0.06). There was no relationship between BMI and GLP-1 for PD subjects (*p* = 0.51, *r* = 0.17, *r*^2^ = 0.03) nor for PD and control subjects combined (*p* = 0.23, *r* = 0.25, *r*^2^ = 0.06) (data not shown).

### Diet and Activity

Diet was assessed in a subset of subjects (*n* = 11 control, *n* = 14 PD) using a validated questionnaire (ASA24^®^) (Subar et al., 2012). There were no significant between group differences in total calories consumed, macro-nutrients, micro-nutrients, or any other food items assessed (*t*-test, all >0.05) with the exception of cured meat which was higher in the PD group (*t*-test, *p* = 0.04) (data not shown). Similarly, activity levels were assessed in a subset of subjects (*n* = 7 control, *n* = 10 PD) self-reported as sedentary, low active, active, very active, and extremely active. Chi Square analysis revealed no significant between group differences in activity level (Chi Square, *p* = 0.33) (data not shown).

## Discussion

Our data demonstrate that compared to healthy subjects, PD patients have a diminished capacity to secrete GLP-1 into the systemic circulation in response to a meal. Data published by our group and others demonstrate that PD patients have an altered intestinal microbiome (i.e., microbial dysbiosis) associated with reduced SCFA levels, and this alteration could contribute to the observed blunted GLP-1 response (Keshavarzian et al., 2015; Unger et al., 2016; Sun and Shen, 2018). Further studies are needed to elucidate the mechanism of diminished GLP-1 in PD patients, and these studies should include an assessment of intestinal L-cell density in PD patients. Future studies should also assess the relationship between GLP-1 and intestinal bacterial populations and their metabolites including SCFA (both influenced by intestinal motility and dietary habits), as well as determine how PD medications may impact GLP-1 levels. For example, prior studies demonstrate a regulatory role of dopamine on appetite which is mediated in part by GLP-1 and by dopaminergic receptors in the amygdala (Alexiadou and Tan, 2020). Thus, it is conceivable that anorexic effects of dopaminergic medications like L-DOPA taken by PD patients might contribute to abnormal GLP-1 homeostasis. However, we do not believe that PD medication-induced anorexia impacted the results in this study, since all subjects consumed the prescribed small meal.

Disrupted secretion of GLP-1 in PD patients may be clinically important, and careful elucidation of causative mechanisms could offer new therapeutic strategies. GLP-1 is known to have neuroprotective properties that are disease-modifying in models of PD (Bertilsson et al., 2008; Kim et al., 2009; Li et al., 2009, 2020; Yun et al., 2018; Fang et al., 2020; Salles et al., 2020; Zhang et al., 2020; Wang et al., 2021). Activation of microglia and the resulting neuroinflammation have been heavily implicated in PD. Microglia in the brain express GLP-1 receptors (Athauda and Foltynie, 2016) and the GLP-1 receptor agonist Exendin-4 decreases microglial activation and protects neurons in a mouse model of PD (Kim et al., 2009). Thus, decreased secretion of GLP-1 in PD patients may be a mechanism contributing to sustained microglial activation and thereby contribute to clinical progression. In our study, however, we did not observe a relationship between GLP-1 and PD clinical characteristics (motor severity/function or disease duration), so the clinical relevance of diminished GLP-1 secretion is not yet clear.

There are some limitations worth noting. Admittedly, this study was powered to detect differences in postprandial GLP-1 secretion between PD and control subjects and was not adequately powered to assess the relationship between clinical characteristics (disease severity or duration) and GLP-1 secretion. Based on comparison of those in the highest and lowest tertials of MDS-UPDRS scores, we predict that future studies will require at least 43 subjects in each group to achieve 80% power to understand the relationship between PD disease characteristics and GLP-1 secretion. In this study, no between group differences in activity, BMI, or diet were observed; however, differences in sex between PD subjects and controls may have contributed to between group differences as sex hormones can influence post prandial GLP-1 secretion (Carroll et al., 2007). It should be noted that differences in sex between PD and control subjects was a consequence of the study design which included household partners as controls to minimize the impact of environmental factors (like diet) on outcome measures. We believe the impact of environmental factors on microbiota (which is an important driver of GLP-1 homeostasis) is more relevant than sex since all female participants in this study were post-menopausal women over the age of 55. However, more women need to be included in future studies so the effects of hormones can be considered. Finally, additional longitudinal studies with larger sample sizes and PD patients with a wide range of disease severities are needed to determine whether GLP-1 is associated with PD clinical characteristics.

The data in this study demonstrate that treated PD patients have blunted postprandial GLP-1 secretion compared to controls. As such, we cannot form conclusions regarding the role of GLP-1 in PD development; however, we have uncovered an interesting relationship between GLP-1 and PD which suggests systemic release of GLP-1 is dysregulated. Metabolic disorders including diabetes, obesity, and metabolic syndrome are increasingly believed to be risk factors for PD (Schernhammer et al., 2011; Zhang and Tian, 2014; Biosa et al., 2018; Pagano et al., 2018; Brauer et al., 2020; Jeong et al., 2020). A common feature among these disorders is low levels of systemic GLP-1 (Ranganath et al., 1996; Bagger et al., 2011; Michalowska et al., 2021). It is intriguing to consider that a blunted GLP-1 response in diabetes, obesity, or metabolic syndrome might lead to increased neuroinflammation and contribute to PD pathogenesis. To date, one study has demonstrated that diabetic patients on GLP-1 agonist treatment have reduced risk of PD, lending credence to this notion (Brauer et al., 2020). Further studies are needed to determine if GLP-1 agonists influence risk of developing PD.

The data in this report provide a compelling scientific rationale for future studies to determine whether augmentation of GLP-1 secretion and GLP-1 signaling are effective therapeutic strategies to prevent and/or delay progression of PD. Indeed, a recent study demonstrated that the probiotic *Clostridium butyricum* (which increases GLP-1 production and upregulates GLP-1 receptor in the brain) improves motor deficits, and reduces dopaminergic neuronal loss in MPTP-treated mice (Sun et al., 2021) suggesting that microbiota-directed treatments may be disease modifying. Currently available therapies to augment GLP-1 signaling, such as GLP-1 receptor agonists and/or microbiota-directed interventions to increase SCFA production, can be rapidly repurposed to test these hypotheses and, if results are positive, be used to treat PD patients.

## Data Availability Statement

The raw data supporting the conclusions of this article will be made available by the authors, without undue reservation.

## Ethics Statement

The studies involving human participants were reviewed and approved by Rush University Medical Center Institutional Review Board (IRB). The patients/participants provided their written informed consent to participate in this study.

## Author Contributions

RM, LV, CG, DH, CF, RV, and AK contributed to conception and design of the study. LV, CG, DH, and AK participated in subject recruitment and assessments. PE, GS, and SR collected samples and data. PE and GS organized the database. RV performed the statistical analysis and created graphs. RM and RV wrote the first draft of the manuscript. PE, LV, CG, DH, SR, and AK wrote sections of the manuscript. All authors contributed to the manuscript revision, read, and approved the submitted version.

## Conflict of Interest

The authors declare that the research was conducted in the absence of any commercial or financial relationships that could be construed as a potential conflict of interest.
